# Carcinoma *in situ* testis displays permissive chromatin modifications similar to immature foetal germ cells

**DOI:** 10.1038/sj.bjc.6605880

**Published:** 2010-09-07

**Authors:** K Almstrup, J E Nielsen, O Mlynarska, M T Jansen, A Jørgensen, N E Skakkebæk, E Rajpert-De Meyts

**Affiliations:** 1 Department of Growth and Reproduction, GR-5064, Rigshospitalet, Blegdamsvej 9, DK-2100 Copenhagen, Denmark

**Keywords:** epigenetics, testicular cancer, carcinoma *in situ*, foetal germ cells, chromatin

## Abstract

**Background:**

The majority of testicular germ cell cancers develop through a pre-invasive carcinoma *in situ* (CIS) stage. The CIS cell is a neoplastic counterpart of foetal germ cells. During their development, foetal germ cells undergo extensive and essential epigenetic modifications, but little is known about epigenetic patterns in CIS cells.

**Methods:**

Immunohistochemistry was used to investigate epigenetic patterns in CIS, germ cell tumours, normal adult and foetal testicular tissue.

**Results:**

CIS cells show low levels of DNA methylation and repressive histone modifications H3K9me2 and H3K27me3, but high levels of H3K9 acetylation, H3K4 methylation and H2A.Z, which all are associated with an activated and accessible chromatin structure. Collectively this renders a permissive chromatin structure and in accordance high levels of RNA polymerase II activity and proliferation (Ki-67 and mitotic index) is observed in CIS cells. Epigenetic patterns similar to that of CIS cells were observed in human gonocytes present within sex cords in foetal testes but correspond to migrating primordial germ cell in mice. Development of overt tumours involves epigenetic repression of the chromatin.

**Conclusion:**

CIS cells have a permissive and foetal-like chromatin structure, which is associated with a high transcriptional and proliferative activity, likely empowering neoplastic transformation. Developmental epigenetic cues in foetal germ cells are substantially different between humans and mice.

Testicular cancer is the most common cancer in young men and the incidence is increasing (Huyghe *et al*, 2003). Genetic predisposition is indicated by familial cases and ethnic differences in incidence rates but the increasing trend and major geographic differences in incidence are consistent with environmental influence. Increased use of endocrine disruptors has been suggested to be one of the environmental factors responsible for the increasing incidence of testicular cancer, which is regarded as one of the outcomes of the testicular dysgenesis syndrome, a group of reproductive disorders linked together by developmental origins (Skakkebaek *et al*, 2001).

The vast majorities of testicular tumours are derived from germ cells and are traditionally divided into a morphologically homogeneous group of seminomas and a very heterogeneous group of non-seminomas. Even though seminomas and non-seminomas histologically are very different, they both originate from the same precursor called carcinoma *in situ* (CIS), intratubular germ cell neoplasia or testicular intraepithelial neoplasia. Thus, CIS has a central role in pathogenesis of testicular cancer.

Evidence from morphological (Nielsen *et al*, 1974; Albrechtsen *et al*, 1982; Skakkebaek *et al*, 1987), epidemiological (Hemminki and Li, 2002; Hemminki *et al*, 2002) immunohistochemical (Rajpert-De Meyts *et al*, 2003; Honecker *et al*, 2004; Pauls *et al*, 2006), and gene expression profiling studies (Almstrup *et al*, 2004; Sonne *et al*, 2009) indicate that the CIS cell is derived from a gonocyte or primordial germ cell (PGC). We have earlier shown that CIS cells are indistinguishable from gonocytes in terms of gene expression profiles (Sonne *et al*, 2009) but the exact developmental time point and the mechanisms of malignant transformation remain unknown. It is possible that a slight delay of gonadal differentiation, under-stimulation of the developing testis or physical misplacement of PGC during migration or colonisation of the gonad might be triggering factors. This will imply that immature germ cells would later become neoplastic due to their undifferentiated nature in the changed microenvironment of the adult testis.

The PGCs and gonocytes are known to undergo extensive epigenetic reprogramming. Seki *et al* (2007) observed that migrating murine PGCs progressively erase H3K9me2 and subsequently establish H3K27me3 concurrently with erasure of genome-wide DNA methylation. Both histone marks are repressive and in the transition between erasure of H3K9me2 and establishment of H3K27me3 PGCs most probably display a transient period with possible hyper-transcription. Hyper-transcription seems however to be prevented as PGCs were shown to enter G2 arrest and repress RNA polymerase II-dependent transcription in this transition period (Seki *et al*, 2007). Hajkova *et al* (2008) showed that mice PGCs upon gonadal colonisation (E11.5) lose most of their linker histone H1, H3K9ac, H3K9me3, H3K27me2 and H4/H2A R3me2.

Very little is known about the epigenetic status of CIS cells, probably because it has not been possible to cultivate CIS cells. Immunohistochemical investigations have shown that CIS cells have arginine 3 of histone H2A and H4 dimethylated (H4/H2A R3me2), which are thought to repress – among others – the *HOX* genes, involved in somatic differentiation programs (Ohinata *et al*, 2005). Most seminomas also have high levels of H4/H2A R3me2, whereas low levels are observed in non-seminomas (Eckert *et al*, 2008). It is also known that the genome of CIS cells contains very little DNA methylation (Netto *et al*, 2008), in contrast to highly methylated non-seminomas, whereas seminomas display variable but mainly low levels of DNA methylation (Smiraglia *et al*, 2002; Netto *et al*, 2008). This is in accordance with our findings of high expression of the DNA methylation enzymes DNA methyl transferase 3A and DNA methyl transferase 3L in the undifferentiated non-seminoma component, embryonal carcinoma (EC), when compared with seminoma and CIS (Almstrup *et al*, 2005).

As developmental epigenetic cues seem a prerequisite for proper germ cell development in mice, we, in this study, addressed the question whether early human neoplastic germ cells – the CIS cells – that seem developmentally arrested, indeed could have an epigenetic status similar to immature germ cells. We also hypothesised that epigenetic modifications may be associated with differences in transcriptional activity between normal and malignant germ cells. To answer that, we systematically investigated the pattern of DNA methylation and histone modifications, which are known to be differentially set during embryonic development of the germ line, in CIS cells and derived cancers and compared them to the epigenetic patterns of the same modifications in normal human germ cells in the adult testis and in foetal gonocytes. We also assessed the transcriptional activity and proliferation index in the CIS cells.

## Materials and methods

### Tissues

The regional Committee for Medical Research Ethics in Denmark approved the use of human tissues stored in the tissue archives of Copenhagen University Hospital. The tissues were obtained after orchidectomy for testicular cancer (adult testis and testicular tumours) from spontaneous abortions/miscarriages (foetal tissues) and from control biopsies to monitor for relapses of acute leukaemia (pre-pubertal testis samples). Tissue samples were fixed overnight at 4°C in buffered formalin or 4% paraformaldehyde. Histological diagnosis was established by experienced pathologists and confirmed by immunohistochemical staining for tumour markers (as described below).

### Immunohistochemical evaluation of DNA methylation and histone modifications

Immunohistochemistry was performed as previously described (Rajpert-De Meyts *et al*, 2003). A standard indirect peroxidase method was used and development was done primarily with 3-amino-9-ethylcarbazole (yielding a red colour). For double staining, colour development with 5-bromo-4-chloro-3-indolyl phosphate and nitro blue tetrazolium was in addition applied (yielding a deep blue colour) as previously described (Jørgensen *et al*, 2009).

The following antibodies were used at indicated dilutions and buffers for microwave treatment: anti-5-methylcytosine, 1:400, (TEG buffer) TRIS 1.21 g/l, EGTA 0.19 g/l, pH 9.0 (5-meC; Calbiochem, Darmstadt, Germany, NA81), rabbit anti-human placental alkaline phosphatase, 1:300, TEG buffer (placental-like alkaline phosphate; Thermo Fisher Scientific, Waltham, MA, USA, RM-9115-S0), anti-dimethyl-histone H3 (Lys9) 1:250, TEG buffer (H3K9me2; Millipore, Billerica, MA, USA, 07–441), anti-trimethyl-Histone H3 (Lys27), 1:500, citrate buffer (H3K27me3; Millipore, 07–449); anti-histone H3 (mono methyl Lys4), 1:6000, TEG buffer (H3K4me1; Abcam, Cambridge, MA, USA, ab8895), anti-histone H3 (di+tri methyl Lys4), 1:2000, TEG buffer (H3K4me2/3; Abcam, ab6000), anti-EZH2, 1:2500, urea buffer (Millipore, 07–689), anti-UTX, 1:50, urea buffer (Abcam, ab36938), anti-JMJD3, 1:75, citrate buffer (Millipore, 07–1533), anti-histone H3 (acetyl Lys9), 1:10000, citrate buffer (H3K9ac; Abcam, ab10812), anti-histone H2A.Z 1:3000, citrate buffer (Abcam, ab4174), anti-RNA polymerase II (N-20), 1:500, citrate buffer (Santa Cruz Biotechnology, Santa Cruz, CA, USA, sc-899), anti-RNA polymerase II Ser-2 phosphorylated, 1:200, TEG buffer (H5; Covance, Princeton, NJ, USA, MMS129R), anti-OCT4 (C-10), 1:250, urea buffer (Santa Cruz Biotechnology, sc5279). For negative controls, serial sections were processed with the primary antibody replaced by the dilution buffer alone. None of the control slides showed any staining (data not shown). Stained slides were scanned on a NanoZoomer (Hamamatsu Photonics, Herrsching am Ammersee, Germany) and analysed using the software NDPview (Hamamatsu Photonics). For each antibody at least three different samples of the same histology were investigated. Intensity of the staining, in cells from the histology in question, was evaluated and scored from negative (neg) to strong positive (+++) as outline in Table 1.

### Proliferation index

A total of 13 tissue samples containing CIS (no overt tumours) and four normal adult testes (patients with obstructive forms of infertility) were included. The proliferation rate was assessed by two methods: the mitotic index and the Ki-67-labelling index and proliferation rates of CIS cells were compared with those of normal spermatogonia. Biopsies were fixed in formalin or Stieve's fixative and stained with haematoxylin and eosin or by immunohistochemistry for placental alkaline phosphatase (mouse anti-human placental alkaline phosphatase; placental-like alkaline phosphate; Dako, Glostrup, Denmark, M7191) to identify CIS cells, and Ki-67 (Dako, M7189) to mark proliferating cells.

Statistical analysis was conducted by Mann–Whitney *U*-test to compare the median of two unmatched groups and Kruskal–Wallis test to compare averages of more than two samples.

## Results

### DNA methylation levels in CIS cells

**Figure 1:**
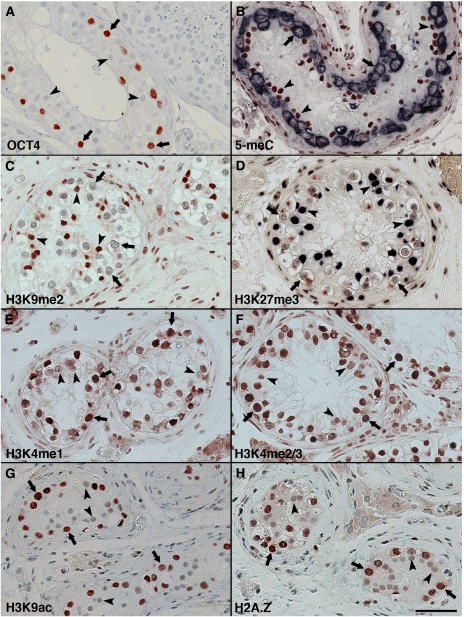
Epigenetic patterns in carcinoma *in situ* (CIS) testis visualised by immunohistochemistry. (**A**) A typical tubule with CIS cells marked by one of the classical markers, OCT4 (POU5F1) (de Jong *et al*, 2005; Rajpert-De Meyts *et al*, 2004). Note that CIS cells are bigger than unstained spermatogonia (visible in the tubule on the right) and have large irregular nuclei with coarse chromatin clumps. (**B**) Double staining for 5-methyl-cytosine (reddish brown) and the classical CIS marker placental-like alkaline phosphate (PLAP, dark blue). (**C**) H3K9me2. (**D**) Double staining for H3K27me3 (deep blue) and PLAP (reddish brown). (**E**) H3K4me1, (**F**) H3K4me2/3, (**G**) H3K9ac and (**H**) H2A.Z. Arrows denote CIS cells and arrowheads Sertoli cells. Bar represent 50 microns.

We first verified results from earlier work on the level of DNA methylation as measured by cytosine methylation (Smiraglia *et al*, 2002; Netto *et al*, 2008). The classical cytoplasmic CIS marker, placental-like alkaline phosphate, was used simultaneously with staining for 5-methyl-cytosine and indeed verified low levels of DNA methylation in CIS cells (Figure 1B). We also confirmed that non-seminomas have high levels of DNA methylation, whereas seminomas show variable but predominantly low DNA methylation levels (data not shown).

### Histone modifications in CIS cells

Immunohistochemical staining for the repressive chromatin modifications H3K9me2 and H3K27me3 revealed low levels of these modifications in CIS cells, whereas Sertoli cells in CIS-containing tubules showed high levels of both modifications (Figure 1C and D).

Activating modifications H3K4me1 and H3K4me2/3 were observed abundant in CIS cells, whereas a weak staining was observed in Sertoli cells (Figure 1E and F). Furthermore, H3K9ac as well as H2A.Z were strongly positive in CIS cells but present at a much lower level in Sertoli cells (Figure 1G and H).

### Histone modifications in overt germ cell tumours

Seminomas showed high levels of H3K9me2, H3K27me3, H3K4me1 and H2A.Z, whereas low levels of H3K4me2/3 and H3K9ac were observed (Supplementary Figure S1). Some variation was observed for the H3K9me2 mark between seminoma samples (from a faint staining to strongly positive).

The undifferentiated component of non-seminomas, EC, showed no H3K27me3 and H3K9me2 staining but moderate levels of H3K4me2/3 methylation. A weak staining was observed for H3K4me1, H3K9ac and H2A.Z in EC (Supplementary Figure S1).

### Histone modifications in normal adult testis

In normal testicular tissue H3K9me2 showed a very specific signal in a subset of spermatogonia, probably type A, and in round and elongating spermatids, whereas Sertoli cells was only faintly stained (Supplementary Figure S1), in contrast to the strongly stained Sertoli cells in CIS tubules (Figure 1C).

H3K27me3 by contrast was present in a broader set of germ cells with a low level in most spermatogonia and higher expression in round to elongating spermatids. Sertoli cells show high levels of H3K27me3 in both normal and CIS-containing tubules (Supplementary Figure S1 and Figure 1D).

H3K4me1 was observed in high levels in a subset of spermatogonia, probably type B but was also weakly present in other types of spermatogonia and Sertoli cells. Expression was again observed in late round spermatids but not in elongated spermatids. However, H3K4me2/3 was observed in a broad range of germ cells including elongating spermatids and in Sertoli cells (Supplementary Figure S1).

H3K9ac was abundant in spermatogonia, present at lower levels in spermatocytes but was absent from late spermatids. A low level was observed in Sertoli cells. Expression of H2A.Z was similar to the observed levels of H3K9ac, except that H2A.Z was highly expressed in round spermatids (Supplementary Figure S1).

### Histone modifications in human foetal and pre-pubertal germ cells

**Figure 2:**
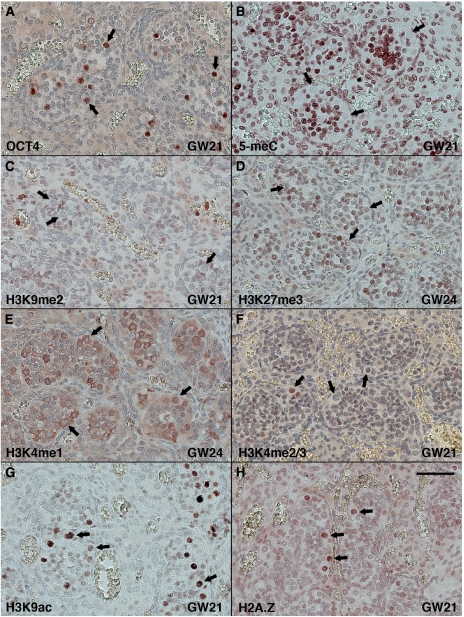
Histone modifications in human foetal gonads. Fetal testis tissues at GW 21–24 marked by immunohistochemical staining for (**A**) OCT4 (POU5F1) to visualise gonocytes, (**B**) 5-methylcytosine (5-meC), (**C**) H3K9me2, (**D**) H3K27me3, (**E**) H3K4me1, (**F**) H3K4me2/3, (**G**) H3K9ac and (**H**) H2A.Z. Arrows indicate foetal germ cells. Bar represents 50 microns.

Samples of foetal gonad tissue ranging from gestation week 17 to 41 were investigated to explore the level of histone modifications in human foetal germ cells. Foetal germ cells around GW 21–24 showed no DNA methylation and absence of repressive H3K9me2, H3K27me3 modifications (Figure 2B–D). In contrast, H3K9ac and H2A.Z were very abundant in foetal germ cells (Figure 2G and H). Surprisingly, H3K4me1 was observed in the cytoplasm of foetal germ cells and H3K4me2/3 was observed in high levels in only a subset of the foetal germ cells (Figure 2E and F). It was roughly estimated that 20% of the foetal germ cells around GW 21 were marked with H3K4me2/3 with slight variations in the amount of positive foetal germ cells observed at earlier and later time points. However, no definitive trend could be identified.

The level of the investigated histone modifications did not differ significantly between foetal and adult Sertoli cells except for H3K9me2, which was found in high levels in Sertoli cells surrounding CIS and in foetal germ cells but not in normal Sertoli cells of any age (Figures 1C, 2C and supplementary Figure S1).

All results are outlined in Table 1.

### Regulation of the H3K27me3 mark in CIS cells

Demethylation of di- and tri-methylated histone H3 at K27 can be carried out by the Jumonji proteins UTX and JMJD3 (Agger *et al*, 2007), whereas methylation of H3K27 can be carried out by Enhancer of Zeste homologue 2 (EZH2), the catalytically active component of the Polycomb repressive complex 2 (Cao *et al*, 2002; Czermin *et al*, 2002).

We observed that UTX and JMJD3 were not expressed in CIS cells (Supplementary Figure S2B and C) but surprisingly, EZH2 was highly expressed in the cytoplasm of CIS cells (Supplementary Figure S2A). JMJD3 was weakly expressed in Sertoli cells, whereas only a faint EZH2 staining was observed in the cytoplasm of Sertoli cells. In overt tumours, faint staining of both proteins was observed, except for EZH2 expression in EC, which was totally absent (data not shown).

Re-evaluation of earlier genome-wide studies of gene expression in CIS cells (Almstrup *et al*, 2004; Skotheim *et al*, 2005; Sonne *et al*, 2009) did not indicate that any of the Polycomb genes or other transcripts encoding for proteins known to be involved in modifying histones were differentially expressed in CIS cells when compared with normal testicular tissue or gonocytes.

### Transcriptional activity and proliferation of CIS cells

**Figure 3:**
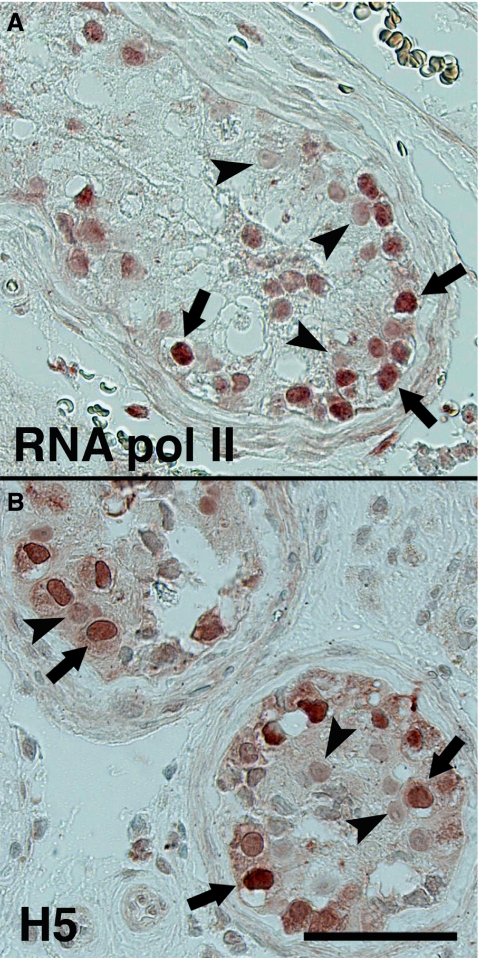
RNA polymerase II activity in CIS cells. Immunohistochemical detection of RNA polymerase II (**A**) and its Ser2-phosphorylated variant as measured by the H5 antibody (**B**). Arrows denotes CIS cells and arrowheads Sertoli cells. Bar represent 50 microns.

Methylation of histone H3K9 demarcates heterochromatin, whereas H3K4 methylation demarcates euchromatin (Fischle *et al*, 2003; Li *et al*, 2008) and collectively the above histone modifications observed in CIS cells could indicate an ‘open’ and permissive chromatin structure in CIS cells. We thus investigated the levels of RNA polymerase II and its Ser2-phosphorylated variant as measured by the H5 antibody. Ser2 phosphorylation is tightly associated with transcriptional elongation (Phatnani and Greenleaf, 2006) and is a marker of the global transcriptional activity. Both RNA polymerase II and its Ser2-phosporylated variant were present in CIS cells in high levels (Figure 3A and B) indicating that transcription is highly active in CIS cells.

The RNA polymerase II was also highly expressed in a subset of spermatocytes and spermatogonia, in seminomas, but was, surprisingly, lower expressed in EC than CIS. High expression of RNA polymerase II, however, did not always correlate with a strong H5 antibody staining. In seminomas, the H5 level was low and expression of RNA polymerase II was high, whereas both RNA polymerase II itself and its active Ser2 form were observed in moderate levels in EC (data not shown).

These data suggested that CIS cells are highly transcriptionally active, which could reflect a high proliferation rate. Assessment of the proliferation rate of CIS cells was thus investigated by counting the mitotic index and the Ki-67-labelling index on 13 tissue sections containing CIS. Comparison of both mitotic index and Ki-67-labelling index of CIS cells and spermatogonia indeed indicated that CIS cells are highly proliferative. A statistically significant difference (*P*=0.001) was observed between the mitotic index and Ki-67-labelling index of normal spermatogonia and those of the CIS cells (Table 2). No significant differences in the proliferation ratios were observed between the CIS cells adjacent to seminomas compared with CIS cells adjacent to non-seminomas (data not shown).

## Discussion

In this study, we performed a detailed analysis of epigenetic status of germ cell neoplasia and show that the precursor for the majority of germ cell cancers – carcinoma *in situ* – possesses low levels of repressive chromatin modifications (H3K9me2 and H3K27me3) concurrently with low DNA methylation and a range of activating chromatin modifications (H3K4me, H3K9ac, and H2A. Z). We also show that RNA polymerase II seems to be very active in CIS cells, and that this fits with a high proliferation rate of CIS cells.

On the basis of a high similarity between gene expression profiles of CIS cells and gonocytes we have earlier suggested that the CIS cell is a transformed gonocyte (Sonne *et al*, 2009). The present data further substantiate that hypothesis as the epigenetic profiles of gonocytes and CIS are highly similar but markedly differ from those of adult germ cells. The only exception was the level of H3K4me1 and H3K4me2/3 that were both abundantly present in the CIS nucleus but had only a limited presence in foetal germ cells. H3K4me2/3 was observed only in a subset of foetal germ cells, most likely gonocytes, which at the gestational age in the samples available for the study constitute only a very small minority of foetal germ cells. Identification of histone variants in the cytoplasm often indicates that they are bound for degradation by chaperones but the importance of the apparent discrepancy between foetal germ cells and CIS is currently unknown. Methylation of histone H3 at Lys4 is, however, known to be tightly associated with methylation of DNA. During embryonic development RNA polymerase II binds to CpG islands and directs H3K4 methylation by recruitment of H3K4 methyltransferases (Guenther *et al*, 2007). Such CpG regions are consequently methylated at Lys4 of histone H3, whereas the rest of the genome contains unmethylated H3K4. In fact, DNA methyl transferase 3L is known to interact directly with histone H3 when unmethylated at Lys4 and facilitate *de novo* methylation. Consequently, DNA methylation may be prevented at such CpG islands primed by RNA polymerase II due to methylation of H3K4. This is in line with the correlation between the high levels of H3K4 methylation and the low DNA methylation levels observed in CIS cells. In addition, the high RNA polymerase II levels could be a consequence of the correlation between RNA polymerase occupancy, H3K4 methylation and low DNA methylation. Similarly, it is known that H2A.Z protects genes from DNA methylation (Zilberman *et al*, 2008), so the high H2A.Z expression in CIS cells is consistent with their low DNA methylation. Loci containing H2A.Z have been observed predominantly at sites occupied by RNA polymerase II along with enhancer regions (Barski *et al*, 2007) and again this seems to fit with the observed high levels of elongating RNA polymerase II in CIS cells. However, we do not know whether the DNA methylation in CIS cells may be hydroxylated and thus unrecognised by the currently used antibodies (Tahiliani *et al*, 2009; Huang *et al*, 2010). Low DNA methylation in the gene body and out of a CpG context has recently been suggested to be of great importance in embryonic stem cells (Ramsahoye *et al*, 2000; Lister *et al*, 2009).

In mice, during PGC migration a progressive erasure of H3K9me2 and establishment of H3K27me3 occurs concurrently with genome-wide erasure of DNA methylation (Seki *et al*, 2007). In addition, as the PGC colonise the gonad in mice, a range of other epigenetic events occurs, which includes erasure of both H2A.Z and H3K9ac (Hajkova *et al*, 2008). One of the important findings of our study is that the regulation of these chromatin marks is substantially different in humans. Migration of human PGCs takes place during GW 4–6 and after GW 6 germ cells are observed in the gonadal anlage and begin to be surrounded by immature Sertoli cells. From that point of time, the term gonocyte is used to describe the germ cells in the male gonad (Fujimoto *et al*, 1977). At GW 21–24, human gonocytes are definitively post-migrational germ cells but show low levels of H3K27me3 and high levels of H3K9ac and H2A.Z, which is comparable with migrating mouse PGC. In addition, human foetal germ cells at GW 16–24, show absence, or very low levels, of H3K9me3 (Bartkova *et al*, 2010). High levels of H4/H2A R3me2s have also been reported in human GW 19 gonocytes (Eckert *et al*, 2008), matching the epigenetic profile of migrating PGCs in mice (Seki *et al*, 2007; Hajkova *et al*, 2008). There is a possibility that all of these marks are transiently reverted in mouse gonocytes at a later foetal age, however, it is not likely, given that the subset of murine germ cells, recognised later as stem spermatogonia, do retain some features of PGC, including high expression of pluripotency genes.

Presumably there is a tight connection between the epigenetic and DNA repair machinery (Hajkova *et al*, 2008) and previous studies has demonstrated that CIS cells and foetal germ cells use different machinery of cell cycle and DNA repair than that operating in post-pubertal germ cells (for review, see Bartkova *et al*, 2007). This is clearly linked to the post-pubertal switch to meiotic division, which requires double breaks of DNA and their subsequent repair in the process of homologous recombination. The similarities between the epigenetic profile of CIS and foetal germ cells are in concert with these data.

Taken together our results clearly indicate that the CIS genome seems to have a open and foetal-like chromatin structure, which potentially could lead to chromosomal instability and subsequently a malignant phenotype, as observed in some somatic cancers, for example, colorectal cancer (Rodriguez *et al*, 2006). High proliferation rates of CIS cells could be a consequence of the open chromatin in CIS cells but stimulation by gonadotropins and androgens, which normally induce proliferation and maturation of germ cells in the post-pubertal testis, may also have a role. It is currently unknown whether the epigenetic profile observed in CIS remains the same or dynamically changes during malignant progression.

Enhancer of Zeste homologue 2 is one of the key enzymes involved in creating the H3K27me3 mark, and we observed it to be highly expressed in the cytoplasm of CIS cells. This enzyme is known to be amplified in a large range of cancers (Bracken *et al*, 2003) and in prostate cancer it is involved in promoting proliferation and invasiveness (Bryant *et al*, 2007). In benign prostate epithelial cells, EZH2 is expressed at low levels in the cytoplasm but becomes overexpressed in prostate cancer cells, in which its putative function is regulation of actin polymerisation (Su *et al*, 2005; Bryant *et al*, 2008). However, it is not known whether EZH2 is involved in actin regulation in CIS cells or whether the cytoplasmic location is a result of a defective translocation to the nucleus, but EZH2 is most probably not engaged in its ‘normal’ function as part of polycomb or swi/snf complexes as none of the genes encoding co-enzymes were observed overexpressed in CIS.

In contrast to CIS, overt germ cell tumours derived from CIS cells seem to have acquired variable epigenetic modifications. Seminomas show high levels of selected repressive modifications, exemplified by H3K9me2 and H3K27me3. Non-seminomas, however, show high methylation levels (Smiraglia *et al*, 2002; Netto *et al*, 2008), but the embryonal carcinoma, which is the most undifferentiated component of non-seminomas, nevertheless retains a very open and foetal-like histone profile. We observed some heterogeneity, but many more samples need to be investigated to deduce whether, for example, there are discrete subsets of seminomas with different modification patterns. Heterogeneity of DNA methylation was indeed observed in seminomas in which 6 out of 33 samples were positive (Netto *et al*, 2008).

Concepts of epigenetic plasticity in cells, which upon niche disturbances stochastically select for epigenetic heterogeneity and consequently, facilitate the ability of cells to acquire abnormal characteristics, have been put forward (Ushijima and Asada, 2009; Feinberg and Irizarry, 2010). These data are in line with this concept and the current views on the pathogenesis of germ cell tumours, which suggest that developmental disturbances of the somatic niche could result in improper maturation of foetal germ cells (Skakkebaek *et al*, 2001). We can add to this hypothesis that developmental delay of germ cell maturation results in preservation of the permissive foetal epigenetic profile, which on hormone stimulation during puberty leads to an aberrant induction of transcription and proliferation, ultimately leading to germ cell cancer later in life.

## Figures and Tables

**Table 1:** Summary of epigenetic patterns in normal and neoplastic testicular tissues

	**Putative function**	**CIS**	**Gonocytes** a	**SEM**	**NonSEM (EC)**	**Normal adult testis**
5-methyl-cytosine	Chromatin repression	neg	neg	neg/++	+++	Sp.gonia: all, Sp.cytes: all, Sp.tids: all
		Sertoli: ++	Sertoli: ++			Sertoli: ++
						
H3K9me2	Chromatin repression	neg	neg	+/+++	neg	Sp.gonia: A +++, Sp.cytes: neg, Sp.tids: round and elongated ++/neg
		Sertoli: +++	Sertoli: ++			Sertoli: neg/+
						
H3K27me3	Chromatin repression	neg/+	neg/+	+++	neg	Sp.gonia: neg/+, Sp.cytes: neg, Sp.tids: round to elongating +++/+
		Sertoli: +++	Sertoli: ++			Sertoli: +++
						
H3K4me1	Chromatin activation	+++	neg/+ (cytoplasma)	++/+++	neg/+	Sp.gonia: B, Sp.cytes: neg, Sp.tids: late round ++
		Sertoli: +	Sertoli: neg			Sertoli: +
						
H3K4me2/3	Chromatin activation	++/+++	−/++	neg/+	+/++	Sp.gonia: ++/+++, Sp.cytes: +/+++, Sp.tids: round +/++
		Sertoli: +	Sertoli: −/+			Sertoli: +/++
						
H3K9ac	Chromatin activation	+++	+++	neg/+	neg/+	Sp.gonia: +++/+, Sp.cytes: ++/neg, Sp.tids: neg
		Sertoli: neg/+	Sertoli: neg/+			Sertoli: +
						
H2A.Z	Chromatin activation	+++	+++	++/+++	neg/+	Sp.gonia: +/+++, Sp.cytes: ++, Sp.tids: round +++
		Sertoli: neg/+	Sertoli: neg/++			Sertoli: +
						
EZH2	H3K27 tri-methylation	+++ (cytoplasma)	Neg	neg/+	neg	Sp.gonia: neg (+ cytoplasma), Sp.cytes: neg, Sp.tids: neg
		Sertoli: neg	Sertoli: neg			Sertoli: neg
						
UTX	H3K27me3 demethylation	neg	Neg	neg/+	neg/+	Sp.gonia: neg, Sp.cytes: neg/+, Sp.tids: neg
		Sertoli: +	Sertoli: neg			Sertoli: neg
						
JMJD3	H3K27me3 demethylation	neg	neg/+	neg/+	neg/+	Sp.gonia: neg/+, Sp.cytes: neg/++, Sp.tids: neg/+
		Sertoli: neg/+	Sertoli: neg			Sertoli: neg/+ (cytoplasma)

Abbreviations: EC; embryonal carcinoma; EZH2, Enhancer of Zeste homologue 2; neg, negative; NonSEM, non-seminoma; SEM; seminoma; Sp.tid, spermatid; Sp.cytes, spermatocytes; Sp.gonia, spermatogonia.

+, faint staining; ++, apparent; +++, strong positive.

a Gestational week 20–40.

**Table 2:** Proliferation index

	**MI (median)**	**Ki-67 (mean)**
CIS	1.58% (*n*=13)	17.42% (*n*=4)
Spermatogonia	0.25% (*n*=4)	6.75% (*n*=4)
CIS *vs* spermatogonia	*P*=0.001	*P*<0.001

Abbreviations: CIS, carcinoma *in situ*; MI, mitotic index.

## References

[bib1] Agger K, Cloos P, Christensen J, Pasini D, Rose S, Rappsilber J, Issaeva I, Canaani E, Salcini A, Helin K (2007) UTX and JMJD3 are histone H3K27 demethylases involved in *HOX* gene regulation and development. Nature 449(7163): 731–734 17713478 10.1038/nature06145

[bib2] Albrechtsen R, Nielsen M, Skakkebaek N, Wewer U (1982) Carcinoma *in situ* of the testis. Some ultrastructural characteristics of germ cells. Acta Pathol Microbiol Immunol Scand A 90(4): 301–303 6889804 10.1111/j.1699-0463.1982.tb00097_90a.x

[bib3] Almstrup K, Hoei-Hansen C, Nielsen J, Wirkner U, Ansorge W, Skakkebaek N, Rajpert-De Meyts E, Leffers H (2005) Genome-wide gene expression profiling of testicular carcinoma *in situ* progression into overt tumours. Br J Cancer 92(10): 1934–1941 15856041 10.1038/sj.bjc.6602560 PMC2361756

[bib4] Almstrup K, Hoei-Hansen C, Wirkner U, Blake J, Schwager C, Ansorge W, Nielsen J, Skakkebaek N, Rajpert-De Meyts E, Leffers H (2004) Embryonic stem cell-like features of testicular carcinoma *in situ* revealed by genome-wide gene expression profiling. Cancer Res 64(14): 4736–4743 15256440 10.1158/0008-5472.CAN-04-0679

[bib5] Barski A, Cuddapah S, Cui K, Roh T, Schones D, Wang Z, Wei G, Chepelev I, Zhao K (2007) High-resolution profiling of histone methylations in the human genome. Cell 129(4): 823–837 17512414 10.1016/j.cell.2007.05.009

[bib6] Bartkova J, Moudry P, Hodny Z, Lukas J, Rajpert-De Meyts E, Bartek J (2010) Heterochromatin marks HP1c, HP1a and H3K9me3, and DNA damage response activation in human testis development and germ cell tumours. Int J Androl, e-pub ahead of print, doi: 10.1111/j.1365-2605.2010.01096.x 10.1111/j.1365-2605.2010.01096.x 20695923

[bib7] Bartkova J, Rajpert-De Meyts E, Skakkebaek N, Lukas J, Bartek J (2007) DNA damage response in human testes and testicular germ cell tumours: biology and implications for therapy. Int J Androl 30(4): 282–291; discussion 291 17573848 10.1111/j.1365-2605.2007.00772.x

[bib8] Bracken A, Pasini D, Capra M, Prosperini E, Colli E, Helin K (2003) EZH2 is downstream of the pRB-E2F pathway, essential for proliferation and amplified in cancer. EMBO J 22(20): 5323–5335 14532106 10.1093/emboj/cdg542 PMC213796

[bib9] Bryant R, Cross N, Eaton C, Hamdy F, Cunliffe V (2007) EZH2 promotes proliferation and invasiveness of prostate cancer cells. Prostate 67(5): 547–556 17252556 10.1002/pros.20550

[bib10] Bryant R, Winder S, Cross S, Hamdy F, Cunliffe V (2008) The Polycomb group protein EZH2 regulates actin polymerization in human prostate cancer cells. Prostate 68(3): 255–263 18095286 10.1002/pros.20705

[bib11] Cao R, Wang L, Wang H, Xia L, Erdjument-Bromage H, Tempst P, Jones R, Zhang Y (2002) Role of histone H3 lysine 27 methylation in Polycomb-group silencing. Science 298(5595): 1039–1043 12351676 10.1126/science.1076997

[bib12] Czermin B, Melfi R, McCabe D, Seitz V, Imhof A, Pirrotta V (2002) *Drosophila* enhancer of Zeste/ESC complexes have a histone H3 methyltransferase activity that marks chromosomal Polycomb sites. Cell 111(2): 185–196 12408863 10.1016/s0092-8674(02)00975-3

[bib13] de Jong J, Stoop H, Dohle G, Bangma C, Kliffen M, van Esser J, van den Bent M, Kros J, Oosterhuis J, Looijenga L (2005) Diagnostic value of OCT3/4 for pre-invasive and invasive testicular germ cell tumours. J Pathol 206(2): 242–249 15818593 10.1002/path.1766

[bib14] Eckert D, Biermann K, Nettersheim D, Gillis A, Steger K, Jäck H, Müller A, Looijenga L, Schorle H (2008) Expression of BLIMP1/PRMT5 and concurrent histone H2A/H4 arginine 3 dimethylation in fetal germ cells, CIS/IGCNU and germ cell tumors. BMC Dev Biol 8: 106 18992153 10.1186/1471-213X-8-106 PMC2613889

[bib15] Feinberg A, Irizarry R (2010) Evolution in health and medicine Sackler colloquium: Stochastic epigenetic variation as a driving force of development, evolutionary adaptation, and disease. Proc Natl Acad Sci USA 107(Suppl 1): 1757–1764 20080672 10.1073/pnas.0906183107 PMC2868296

[bib16] Fischle W, Wang Y, Allis C (2003) Histone and chromatin cross-talk. Curr Opin Cell Biol 15(2): 172–183 12648673 10.1016/s0955-0674(03)00013-9

[bib17] Fujimoto T, Miyayama Y, Fuyuta M (1977) The origin, migration and fine morphology of human primordial germ cells. Anat Rec 188(3): 315–330 900520 10.1002/ar.1091880305

[bib18] Guenther M, Levine S, Boyer L, Jaenisch R, Young R (2007) A chromatin landmark and transcription initiation at most promoters in human cells. Cell 130(1): 77–88 17632057 10.1016/j.cell.2007.05.042 PMC3200295

[bib19] Hajkova P, Ancelin K, Waldmann T, Lacoste N, Lange U, Cesari F, Lee C, Almouzni G, Schneider R, Surani M (2008) Chromatin dynamics during epigenetic reprogramming in the mouse germ line. Nature 452(7189): 877–881 18354397 10.1038/nature06714 PMC3847605

[bib20] Hemminki K, Li X (2002) Cancer risks in second-generation immigrants to Sweden. Int J Cancer 99(2): 229–237 11979438 10.1002/ijc.10323

[bib21] Hemminki K, Li X, Czene K (2002) Cancer risks in first-generation immigrants to Sweden. Int J Cancer 99(2): 218–228 11979437 10.1002/ijc.10322

[bib22] Honecker F, Stoop H, de Krijger R, Chris Lau Y, Bokemeyer C, Looijenga L (2004) Pathobiological implications of the expression of markers of testicular carcinoma *in situ* by fetal germ cells. J Pathol 203(3): 849–857 15221945 10.1002/path.1587

[bib23] Huang Y, Pastor W, Shen Y, Tahiliani M, Liu D, Rao A (2010) The behaviour of 5-hydroxymethylcytosine in bisulfite sequencing. PLoS One 5(1): e8888 20126651 10.1371/journal.pone.0008888 PMC2811190

[bib24] Huyghe E, Matsuda T, Thonneau P (2003) Increasing incidence of testicular cancer worldwide: a review. J Urol 170(1): 5–11 12796635 10.1097/01.ju.0000053866.68623.da

[bib25] Jørgensen A, Nielsen J, Morthorst J, Bjerregaard P, Leffers H (2009) Laser capture microdissection of gonads from juvenile zebrafish. Reprod Biol Endocrinol 7: 97 19747405 10.1186/1477-7827-7-97 PMC2755477

[bib26] Li F, Huarte M, Zaratiegui M, Vaughn M, Shi Y, Martienssen R, Cande W (2008) Lid2 is required for coordinating H3K4 and H3K9 methylation of heterochromatin and euchromatin. Cell 135(2): 272–283 18957202 10.1016/j.cell.2008.08.036 PMC2614271

[bib27] Lister R, Pelizzola M, Dowen R, Hawkins R, Hon G, Tonti-Filippini J, Nery J, Lee L, Ye Z, Ngo Q, Edsall L, Antosiewicz-Bourget J, Stewart R, Ruotti V, Millar A, Thomson J, Ren B, Ecker J (2009) Human DNA methylomes at base resolution show widespread epigenomic differences. Nature 462(7271): 315–322 19829295 10.1038/nature08514 PMC2857523

[bib28] Netto G, Nakai Y, Nakayama M, Jadallah S, Toubaji A, Nonomura N, Albadine R, Hicks J, Epstein J, Yegnasubramanian S, Nelson W, De Marzo A (2008) Global DNA hypomethylation in intratubular germ cell neoplasia and seminoma, but not in nonseminomatous male germ cell tumors. Mod Pathol 21(11): 1337–1344 18622385 10.1038/modpathol.2008.127 PMC4086525

[bib29] Nielsen H, Nielsen M, Skakkebaek N (1974) The fine structure of possible carcinoma-*in-situ* in the seminiferous tubules in the testis of four infertile men. Acta Pathol Microbiol Scand A 82(2): 235–248 4364167 10.1111/j.1699-0463.1974.tb03848.x

[bib30] Ohinata Y, Payer B, O'Carroll D, Ancelin K, Ono Y, Sano M, Barton S, Obukhanych T, Nussenzweig M, Tarakhovsky A, Saitou M, Surani M (2005) Blimp1 is a critical determinant of the germ cell lineage in mice. Nature 436(7048): 207–213 15937476 10.1038/nature03813

[bib31] Pauls K, Schorle H, Jeske W, Brehm R, Steger K, Wernert N, Büttner R, Zhou H (2006) Spatial expression of germ cell markers during maturation of human fetal male gonads: an immunohistochemical study. Hum Reprod 21(2): 397–404 16210381 10.1093/humrep/dei325

[bib32] Phatnani H, Greenleaf A (2006) Phosphorylation and functions of the RNA polymerase II CTD. Genes Dev 20(21): 2922–2936 17079683 10.1101/gad.1477006

[bib33] Rajpert-De Meyts E, Bartkova J, Samson M, Hoei-Hansen C, Frydelund-Larsen L, Bartek J, Skakkebaek N (2003) The emerging phenotype of the testicular carcinoma *in situ* germ cell. APMIS 111(1): 267–278; discussion 278–279 12752272 10.1034/j.1600-0463.2003.11101301.x

[bib34] Rajpert-De Meyts E, Hanstein R, Jorgensen N, Graem N, Vogt PH, Skakkebaek NE (2004) Developmental expression of POU5F1 (OCT-3/4) in normal and dysgenetic human gonads. Hum Reprod 19: 1338–1344 15105401 10.1093/humrep/deh265

[bib35] Ramsahoye B, Biniszkiewicz D, Lyko F, Clark V, Bird A, Jaenisch R (2000) Non-CpG methylation is prevalent in embryonic stem cells and may be mediated by DNA methyltransferase 3a. Proc Natl Acad Sci USA 97(10): 5237–5242 10805783 10.1073/pnas.97.10.5237 PMC25812

[bib36] Rodriguez J, Frigola J, Vendrell E, Risques R, Fraga M, Morales C, Moreno V, Esteller M, Capellà G, Ribas M, Peinado M (2006) Chromosomal instability correlates with genome-wide DNA demethylation in human primary colorectal cancers. Cancer Res 66(17): 8462–9468 16951157 10.1158/0008-5472.CAN-06-0293

[bib37] Seki Y, Yamaji M, Yabuta Y, Sano M, Shigeta M, Matsui Y, Saga Y, Tachibana M, Shinkai Y, Saitou M (2007) Cellular dynamics associated with the genome-wide epigenetic reprogramming in migrating primordial germ cells in mice. Development 134(14): 2627–2638 17567665 10.1242/dev.005611

[bib38] Skakkebaek N, Berthelsen J, Giwercman A, Müller J (1987) Carcinoma-*in-situ* of the testis: possible origin from gonocytes and precursor of all types of germ cell tumours except spermatocytoma. Int J Androl 10(1): 19–28 3034791 10.1111/j.1365-2605.1987.tb00161.x

[bib39] Skakkebaek NE, Rajpert-De Meyts E, Main KM (2001) Testicular dysgenesis syndrome: an increasingly common developmental disorder with environmental aspects. Human Reproduction 16(5): 972–978 11331648 10.1093/humrep/16.5.972

[bib40] Skotheim R, Lind G, Monni O, Nesland J, Abeler V, Fosså S, Duale N, Brunborg G, Kallioniemi O, Andrews P, Lothe R (2005) Differentiation of human embryonal carcinomas *in vitro* and *in vivo* reveals expression profiles relevant to normal development. Cancer Res 65(13): 5588–5598 15994931 10.1158/0008-5472.CAN-05-0153

[bib41] Smiraglia D, Szymanska J, Kraggerud S, Lothe R, Peltomäki P, Plass C (2002) Distinct epigenetic phenotypes in seminomatous and nonseminomatous testicular germ cell tumors. Oncogene 21(24): 3909–3916 12032829 10.1038/sj.onc.1205488

[bib42] Sonne S, Almstrup K, Dalgaard M, Juncker A, Edsgard D, Ruban L, Harrison N, Schwager C, Abdollahi A, Huber P, Brunak S, Gjerdrum L, Moore H, Andrews P, Skakkebaek N, Meyts E, Leffers H (2009) Analysis of gene expression profiles of microdissected cell populations indicates that testicular carcinoma *in situ* is an arrested gonocyte. Cancer Res 69(12): 5241–5250 19491264 10.1158/0008-5472.CAN-08-4554 PMC2869030

[bib43] Su I, Dobenecker M, Dickinson E, Oser M, Basavaraj A, Marqueron R, Viale A, Reinberg D, Wülfing C, Tarakhovsky A (2005) Polycomb group protein EZH2 controls actin polymerization and cell signaling. Cell 121(3): 425–436 15882624 10.1016/j.cell.2005.02.029

[bib44] Tahiliani M, Koh K, Shen Y, Pastor W, Bandukwala H, Brudno Y, Agarwal S, Iyer L, Liu D, Aravind L, Rao A (2009) Conversion of 5-methylcytosine to 5-hydroxymethylcytosine in mammalian DNA by MLL partner TET1. Science 324(5929): 930–935 19372391 10.1126/science.1170116 PMC2715015

[bib45] Ushijima T, Asada K (2009) Aberrant DNA methylation in contrast with mutations. Cancer Sci 101(2): 300–305 19958364 10.1111/j.1349-7006.2009.01434.x PMC11159270

[bib46] Zilberman D, Coleman-Derr D, Ballinger T, Henikoff S (2008) Histone H2A.Z and DNA methylation are mutually antagonistic chromatin marks. Nature 456(7218): 125–129 18815594 10.1038/nature07324 PMC2877514

