# Supplementary figures and images for: Carcinoma in situ testis displays permissive chromatin modifications similar to immature foetal germ cells
Source: Br J Cancer. 2010 Sep 7;103(8):1269–76. doi: 10.1038/sj.bjc.6605880 (PMC2967056; doi:10.1038/sj.bjc.6605880)

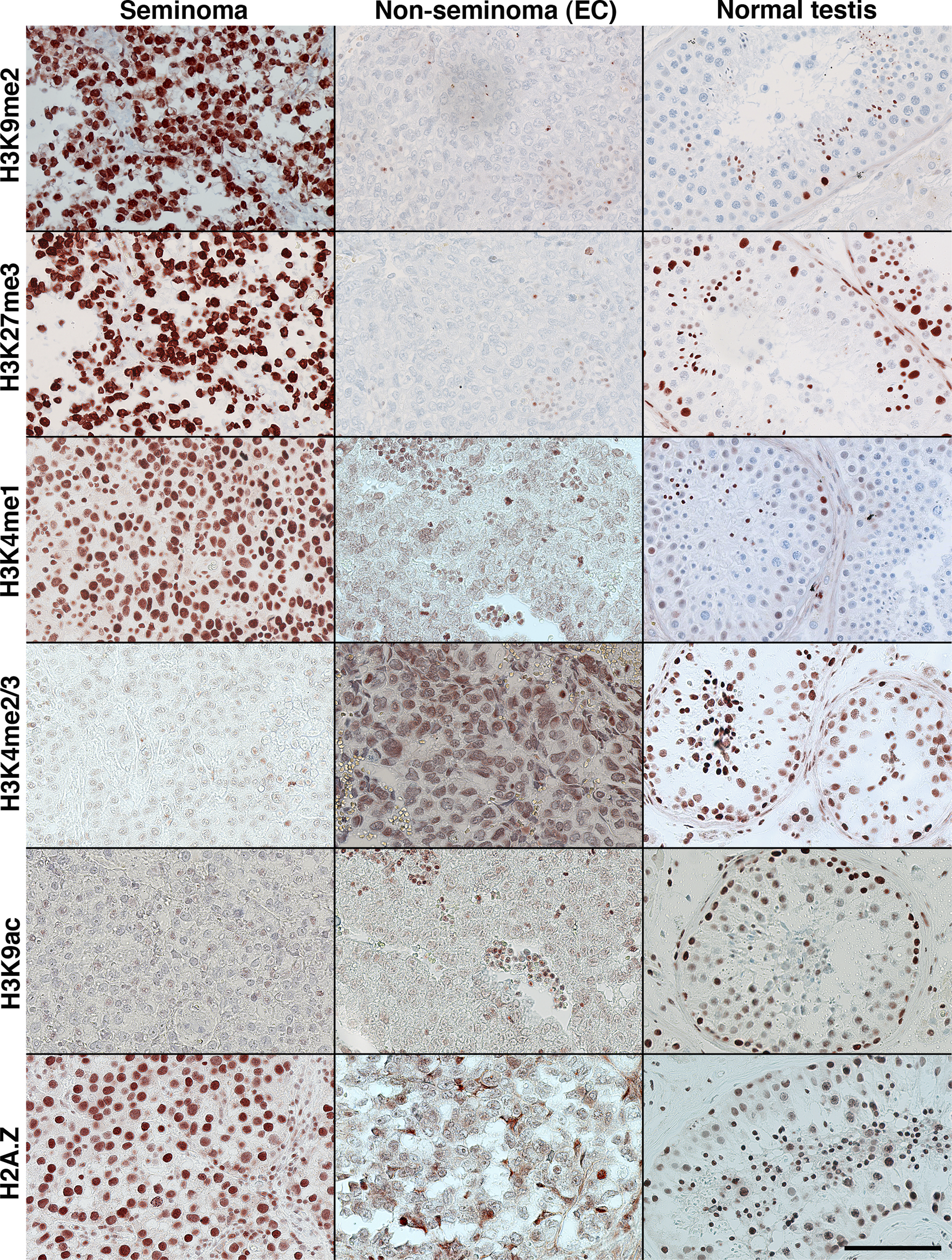

Supplement: Supplementary Figure S1 [file 6605880x1.tif]

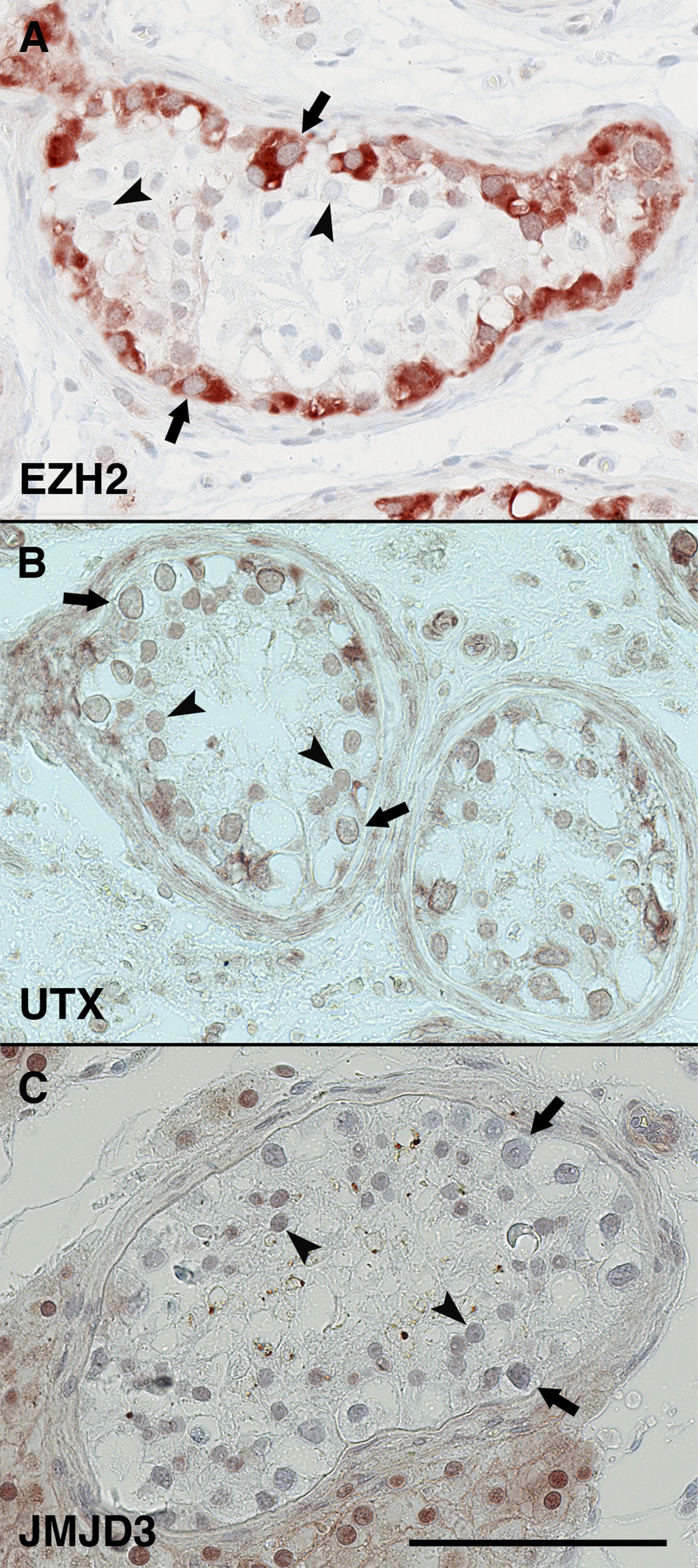

Supplement: Supplementary Figure S2 [file 6605880x2.tif]
