# Supplementary material for: Carcinoma in situ testis displays permissive chromatin modifications similar to immature foetal germ cells
Source: Br J Cancer. 2010 Sep 7;103(8):1269–76. doi: 10.1038/sj.bjc.6605880 (PMC2967056; doi:10.1038/sj.bjc.6605880)
Supplement: Supplementary Figure Legends [file 6605880x3.doc]

**Supplementary figures:**

**Figure S1.** Histone modifications in overt testicular germ cell tumors: seminoma on the left, embryonal carcinoma (the undifferentiated component of non-seminomas) in the middle column and normal adult testis on the right.

See text and Table 1 for detailed description of expression patterns. Bar represent 100 microns.

**Figure S2.** H3K27 methyltransferase and demethylation enzymes in CIS cells.

Immunohistochemical staining for **A)** the H3K27 methyltransferase EZH2, **B)** UTX **B),** and **C)** JMJD3 Arrows denotes CIS cells and arrowheads Sertoli cells. Bar represent 100 microns.
